# Data on impact of technological change on employees' cognitive attitude and organizational performance

**DOI:** 10.1016/j.dib.2018.04.024

**Published:** 2018-04-12

**Authors:** Chinyerem Adeniji, Olufemi Adeyeye, Oluwole Iyiola, Maxwell Olokundun, Taiye Borishade, Hezekiah Falola, Odunayo Salau

**Affiliations:** Covenant University, Nigeria

**Keywords:** Technological change, Employee cognitive attitude, Employee performance, Manufacturing companies, Nigeria

## Abstract

Change is unavoidable for organizations just as it is in every sphere of life. Whatever the reasons are, organizations need to change, keeping in mind the end goal to survive and to be successful. Organizations operate in an environment where globalisation is the common expression of the phenomenon that is driving a great dynamism in the business environment across the world and no business is immune from the effects of this “globalisation”. Competition, policymaking and advancement in technology exist on a day-to-day basis (Hatch, 2009) as well as opportunities are no longer localised within a nation, region or continent, every business is now competing with competitors all over the world. These forces are in constant change and affect a large number of organizations, which involves creating new strategies and policies in order for the organizations to survive and compete within the global business world and also to improve organizational performance but, there are also many challenges as well as the intensification of competition. The usage of technology decides the quality and number of products and services to be delivered. Organizational and national restrictive execution and improvement are controlled by the state and types of technology. Technology likewise impacts the living states of individual and groups in organizations and countries and the relationship between them. Technology is inclined to change, and the condition of technology have direct connection to the relationship between the business and worker. Technology, labour and capital are interconnected. The data presented in this article is very salient in this regard

**Specification table**

**Table:** 

**Subject area**	Business, Management
**More Specific Subject Area:**	Business Administration
**Type of Data**	Table. Figures
**How Data was Acquired**	Researcher-made questionnaire analysis
**Data format**	Raw, analyzed, Descriptive and Inferential statistical data
**Experimental Factors**	Sample consisted of employees of manufacturing companies in Nigeria. The researcher-made questionnaire which contained data technological change on employees’ cognitive attitude and employee performance were completed.
**Experimental features**	Effects of Technological change is a major factor endangering employee performance particularly in the manufacturing sector.
**Data source location**	South west Nigeria
**Data Accessibility**	Data is included in this article

**Value of data**

● The data presented in this article implies that change in firm performance could be as a result of the drastic change in the use and adoption of technology.
● The results suggest that technological change stimulates employees’ cognitive attitude and more attention should be given for employees to have a technical knowledge of the duties they perform.
● The results suggest that technically inclined to the work systems will enhance a positive attitude especially as regards the way employees think thereby ensuring that all necessary activities which foster performance are achieved.

## 1 Data

The Figure and table below shows the predictor importance of technological change dimensions on employees' cognitive attitude and performance of selected manufacturing firms.

### 1.1 Predictor importance of technological change on employees' cognitive attitude and performance of sampled firms

The figure and table above predict the importance of the construct for independent variables on the dependent variables. To assess the coefficient (significant effects) level, regression analysis was adopted as presented in the table below. The level of significance below 0.05 shows the confidence of level of 95%.

**Table:** 

**Model Summary**									
Model	R	R Square	Adjusted R Square	Standard Error of the Estimate	Change Statistics				
					R Square Change	F Change	df1	df2	Significant. F Change
1	.647^a^	.419	0.418	0.58085	.419	303.691	1	421	.000
2	.693^b^	.480	0.478	.55004	.061	49.473	1	420	.000

a. Predictors: (Constant), TECHNOLOGICAL CHANGE

b. Predictors: (Constant), TECHNOLOGICAL CHANGE, EMPLOYEE COGNITIVE ATTITUDE

c. Dependent Variable: ORGANISATIONAL PERFORMANCE

The test was to assess the effect of technological change on employees’ cognitive attitude and performance of selected manufacturing firms. In the first step, the effect of technological change on the performance of selected manufacturing firms was tested. The R-Square value is the degree of variation of the dependent variable which can be predicted by the independent variable. The analysis revealed that technological change accounted for 41.9% variance in firm performance of selected manufacturing firms (*R*^2^=0.419, df (1, 421)=303.691, *p*<0.05). In the second step, the mediating role of employees’ cognitive attitude was examined. The analysis showed that employees’ cognitive attitude was able to explain 48% variance in firm performance over and beyond the effects of technological change (*R*^2^=0.480, df (2, 420)=194.065, *p*<0.05). The significance of the F-change was assessed and it was significant (0.000) as shown in the table below:

**Table:** 

Analysis of Variance						
Model		Sum of Squares	df	Mean Square	F	Significance
1	Regression	102.460	1	102.460	303.691	0.000b
	Residual	142.038	421	0.337		
	Total	244.499	422			
2	Regression	117.428	2	58.714	194.065	0.000c
	Residual	127.070	420	0.303		
	Total	244.499	422			

a. Dependent Variable: ORGANISATIONAL PERFORMANCE

b. Predictors: (Constant), TECHNOLOGICAL CHANGE

c. Predictors: (Constant), TECHNOLOGICAL CHANGE, EMPLOYEE COGNITIVE ATTITUDE

**Fig. 1:**
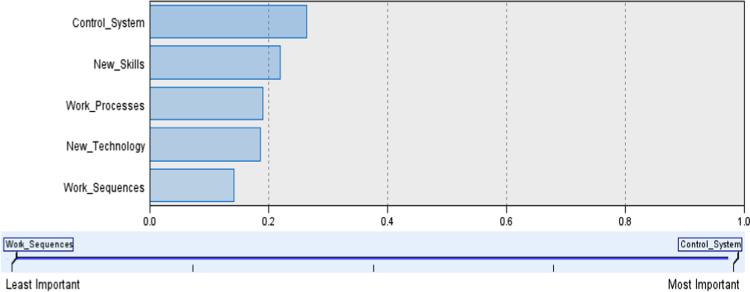
Predictor importance of technological change on firm performance. Source: Researcher's Field Survey Result (2017)

Table above shows the results of the two models. The first model showed the effect of technological change on the performance of selected manufacturing firms. The F-value is calculated as the Mean Square Regression (102.460) divided by the Mean Square Residual (0.337), yielding *F*=303.691. From this results, model 1 in the table is statistically significant (Sig=0.000). The second model examined the effect of technological change on employees’ cognitive attitude and performance of selected manufacturing firms. The *F*-value is calculated as the Mean Square Regression (58.714) divided by the Mean Square Residual (0.303), yielding F= 194.065 at an acceptable significant level of 0.000 (Fig. 1 and Table 1).

**Table:** 

**Coefficients**^**a**^										
Model		Unstandardized Coefficients		Standardized Coefficients	t	Sig.	95.0% Confidence Interval for B		Collinearity Statistics	
		B	Std. Error	Beta			Lower Bound	Upper Bound	Tolerance	VIF
1	(Constant)	1.052	.169	.647	6.240	.000	.720	1.383		
	TECH	.703	.040		17.427	.000	.624	.783	1.000	1.000
2	(Constant)	.763	.165	.439	4.633	.000	.439	1.087		
	TECH	.477	.050		9.559	.000	.379	.575	.586	1.707
	CAT	.288	.041	.323	7.034	.000	.207	.368	.586	1.707

^a^Dependent variable: Organisational performance.

**Table 1:** Predictor Importance of Technological change on employees’ cognitive attitude and performance of Sampled Firms.

**Nodes**		**Importance**
Control_System	Control System	0.26
New_Skills	New Skills	0.22
Work_Processes	Work Processes	0.19
New_Technology	New Technology	0.19
Work_Sequences	Work Sequences	0.14

Based on the results in model 2, the table above revealed the contributions of technological change to employees’ cognitive attitude and organisational performance and their levels of significance. (Change in technology; β = 0.439; t= 9.559; p < .05, employees’ cognitive attitude; β = 0.323; t= 7.034; p < .05).

## 2 Experimental design, materials and methods

Data was gathered from employees’ in selected manufacturing companies with the aid of a researcher- made questionnaire based on the works of [1], [2], [3], [4]. The collected data were coded and entered into SPSS version 22. Data analysis was performed; using SPSS-22 Data was analyzed applying inferential statistical tests which involved regression analysis. Survey research design was adopted for this study where data was collected from a sample size of 600 employees from the three tiers of management of three manufacturing firms in Nigeria namely Cadbury, Plc, Unilever Plc and Seven-up Nigeria, Lagos State to determine the effect of technological change on the cognitive attitude of employees’ and organisational performance. The survey research was adopted and the population of the respondents was made up of 6998 employees. The questionnaire was self-administered to the respondents who willingly filled the research questionnaire. Hierarchical Regression analysis was adopted. The researchers established that the respondents were well informed about the background and the purpose of this research and they were kept up-to-date with the participation process and regime. Every respondent was offered the opportunity to stay anonymous and their responses were treated confidentially. Consent was obtained from the appropriate authorities in the organisations where copies of questionnaire were distributed.
